# Computed tomography localization of radiation treatment delivery versus conventional localization with bony landmarks

**DOI:** 10.1120/jacmp.v4i2.2525

**Published:** 2003-03-01

**Authors:** Albert Y. C. Fung, S.‐Y. Lisa Grimm, James R. Wong, M. Uematsu

**Affiliations:** ^1^ Department of Medical Physics Memorial Sloan‐Kettering Cancer Center 1275 York Avenue New York New York 10021; ^2^ Department of Radiation Oncology Morristown Memorial Hospital 100 Madison Avenue Morristown New Jersey 07962; ^3^ Department of Radiology National Defense Medical College 3–2 Namiki Tokorozawa, Saitama 359 Japan

**Keywords:** localization, computed tomography, portal imaging

## Abstract

A computed tomography (CT) scanner was installed in the linear accelerator room (Primatom) at Morristown. Since June 2000, we have been providing prostate, lung, and liver cancer patients with fusion of CT and linac radiation treatment. This paper describes our registration methods between planning and treatment CT images, and compares treatment localization by CT versus conventional localization by bony landmarks such as portal imaging. For image registration, we printed out beforehand the beam's eye view of the treatment fields. Prostate tumor volume from each Primatom CT slice was mapped on the printouts, and the necessary isocenter shift relative to the skin marks was deduced. No port film was necessary for our Primatom patients. For ten patients we generated digitally‐reconstructed radiographs (DRRs) with bone contrast from the CT scans, and deduced the required shift as the difference between the DRRs of the Primatom CT versus the planning CT This represented the best observable shift should portal imaging be employed. Shift from bony landmark significantly correlated with the Primatom CT shift. Positioning adjustment based on bony anatomy was generally in the same direction as the CT shift for individual patient, but frequently did not go far enough. Our study confirmed that prostate organ motion relative to the bones has an average length of 4.7 mm (with standard deviation of 2.7 mm), and indicated the superiority of CT versus conventional bony structure (such as portal imaging) localization.

PACS number(s): 87.53.Kn, 87.53.–j

## INTRODUCTION

Radiation therapy is usually delivered with preplans and treatment localization at the linear accelerator using conventional portal imaging, film or electronic. This has two disadvantages: (i) Portal image localization is based on bony anatomy which ignores organ motion, and the potentially substantial tumor movement relative to the surrounding bones.^1^ ^–^ ^4^ (ii) Port images are taken with megavoltage x‐ray with image quality far inferior to kilovoltage images. There have been several attempts to improve on the localization method before treatment. The effort of the radiation oncology community included radio‐opaque implanted markers^4^ and ultrasound localization.^5^

**Figure 1:**
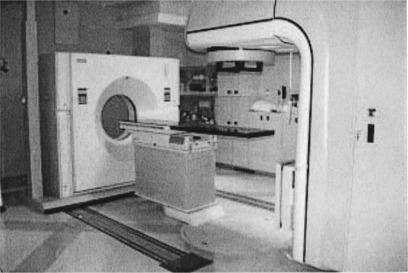
(Color) CT scanner in the same room as a linear accelerator, sharing a single patient couch.

The above two problems can be solved at once by having a computed tomographic (CT) scanner and a linear accelerator in the same room. We have installed this kind of CT scanner in a linear accelerator room at Morristown Memorial Hospital (Fig. 1). Since June 2000 we have been providing prostate, lung, and liver cancer patients with fusion of CT and linac (FOCAL) radiation treatment.^6^ ^–^ ^8^ The major innovation here is that during each radiation fraction, the patient has a scan with CT to locate the tumor, and is then being treated while lying on the same couch without moving. The system, manufactured by Siemens Medical Systems, is called Primatom (Primus, Siemens linear accelerator model, plus Somatom, Siemens CT scanner). The purpose of this paper is to describe our image registration method for FOCAL irradiation, and compare the accuracy of FOCAL versus treatment localization by portal imaging.

## METHODS

The typical prostate prescription at Morristown was in three phases: (I) 45 Gy to the seminal vesicles and the prostate, then (II) 16.2 Gy cone down prostate only, then (III) 10 Gy cone down prostate with FOCAL. Fractionations for the three phases are: (I) daily dose of 180 cGy×25 fractions, (II) 180 cGy×9 fractions, and (III) 200 cGy×5 fractions. We follow the ICRU 50 conventions.^9^ The patients are treated supine, and the treatment beam energy used is 15 MV x‐ray. Treatment planning is performed with the Helax system (Helax treatment planning system, MDS Nordion, Canada). CT scans are performed before phase I planning and repeated before phase II. The Phase II CT is also used in planning phase III irradiation. The clinical target volume (CTV) of phase I includes microscopic disease around the seminal vesicles and the prostate, and conformal four‐field box is administered. The phase II CTV includes only the prostate. By excluding the seminal vesicles, we reduce further dose to the large and small bowels. Phase II is given by intensity modulated radiation therapy (IMRT), which consists of five fields: RPO, RAO, AP, LAO, and LPO. Planning target volume (PTV) coverage, represented by V100, the percentage of PTV covered by at least 100% of prescribed dose, usually exceeds 95%. On the dose volume histograms (DVHs) of the critical organs, less than 30% of the rectum typically receives 90% of prescribed dose (i.e., V90<30%), and less than 50% of the bladder receives 50% of prescribed dose (i.e., V50<50%). The IMRT plan is evaluated separately from the two non‐IMRT plans.

The phase III FOCAL treatment made use of the Primatom, which consisted of a linear accelerator (Primus) and a CT scanner (Somatom) sharing a common patient couch in the same room. During each scanning, the CT gantry slides on a rail while the table top stays motionless. After CT the patient couch is rotated 180° for linear accelerator treatment. The Primatom system was thoroughly tested^8^ with Rando phantom studies to investigate the effectiveness and to estimate the accuracy achievable in using the sliding CT scanner for setup localization. The Rando phantom has a mass of 74 kg, and a weight distribution along the couch similar to a live patient. A quality assurance phantom was also designed to check the mechanical integrity of the sliding gantry CT scanner against slippage and the accuracy of the reconstruction algorithm.^8^ The FOCAL phase was given with three conformal fields (anterior, right lateral, and left lateral) using multileaf collimators (MLCs) to spare the rectum from excessive dose. The PTV had a margin of 1.0 cm around the CTV, except 0.5 cm on the posterior side for additional sparing of rectum. We were confident to give a tight margin on the posterior side due to our ability of visualizing the prostate on the treatment CT Therefore, the margin needed only to account for intrafractional organ motion, with which 0.5 mm was judged adequate for prostate based on measured data in the literature.^1^ ^–^ ^4^ The MLCs had an additional margin of 0.8 cm around the PTV to allow for the beam penumbra of 15 MV x‐ray. Before treatment each patient had a CT scan used in planning (the planning CT), and on each day of FOCAL treatment the patient received a new CT scan at the linear accelerator (the treatment CT). When this protocol was started, we did not want to mix two complicated procedures (IMRT and FOCAL) together; otherwise, any problems would be more difficult to solve. We do have plans to combine IMRT and FOCAL in the future.

**Figure 2:**
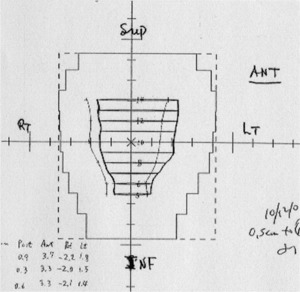
Mapping of prostate contours on treatment day onto planning AP beam's eye view. The solid outline represents the original prostate position (CTV) as in planning CT. The thin outline represents the new prostate position on treatment day relative to (uncorrected) MLC aperture.

Our image registration method between planning and treatment CT scans was as follows. We printed out beforehand on paper the beam's eye view (BEV) MLC shapes of the treatment fields and the planning target volume. After images were captured from the Primatom CT, we first set the origin as the isocenter defined by spherical radio‐opaque markers (BBs) placed on the skin tattoos indicating the entrance points of the central axes of the orthogonal beams. This point was registered with the center on each BEV. Then from the most superior to the most inferior slices containing prostate, the coordinates of the four (anterior, posterior, right, left) extreme borders of the prostate tumor volume (CTV) and the anterior rectal border on each CT slice were recorded and mapped on the BEV printouts (Fig. 2), i.e., the tumor coordinates were measured with Primatom software, and then drawn on paper with the MLCs. The manual transfer of coordinates might cause residual measurement uncertainty, but it was within 0.5 mm, and was not taken into account. The mapped points joined together represented the prostate and rectum location as seen from BEV relative to the MLCs (before shift) on that day. The different BEV projections were consistent with one another most of the time, and the necessary isocenter shift to center the MLCs on the prostate was determined by a physician. Since the gantry isocenter was fixed, the required couch (and prostate) shift would be exactly opposite the isocenter shift (relative to the prostate) shown on the BEVs.

**Figure 3:**
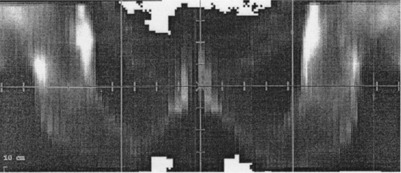
Digitally reconstructed radiograph from treatment CT, used to determine isocenter shift vs planning position.

We chose ten prostate patients randomly for the current study. Random selection of patients was to ensure unbiasedness. After a Primatom CT scan, the patient gained no benefit of taking a port film, therefore no port film was available for our Primatom patients. To find the isocenter shifts that came from bony structure localization technique, we generated digitally reconstructed radiographs (DRRs) with bone contrast from the CT scans (Fig. 3), and deduced the required shifts as the difference between the DRRs of the treatment CT versus the planning CT The DRRs were generated with much better contrast than the portal images, and represented the most accurate shift that could possibly be observed should portal imaging be employed. A separate study to compare DRRs and portal images will be scientifically valuable. Image registration and isocenter shift (i.e., the time delay between CT scan and treatment delivery) took about 15 min for each patient. We did not repeat the CT routinely after adjustment since it would cause further delay before we could verify the new position. Since opposed lateral beams gave redundant information, we just used the left lateral BEV for registration and not the right lateral. As mentioned above, the different views were mostly consistent with one another.

## RESULTS

We have established the correspondence between CT isocenter and machine isocenter in a separate article.^8^ The CT scans right before radiation treatment provided the best knowledge of the position of tumor volume, and were taken as benchmark positions for the purpose of our study. For every patient, let the orthogonal isocenter shifts indicated by the Primatom CT be $p_{i}\;(i=x,y,z)$, and the shifts indicated by bony DRRs be $b_{i}$ Then we could define $s_{i}=p_{i}-b_{i}$ as the shortfall of bony registration, i.e., the prostate organ motion relative to surrounding pelvic bones, or the additional shift required to bring the bone‐shifted position to the CT‐indicated position. Shift data for the ten patients are listed in Table I.

**Table I:** Shift data for the ten patients. Shifts in each orthogonal direction are listed: S/I, R/L, A/P means superior/inferior, right/left, anterior/posterior respectively, and positive shift means towards superior, right, anterior, respectively. The “lengths” are the magnitude of the shift vectors in mm.

Patient		*p*	*b*	*s*	*p*‐length	*b*‐length	*s*‐length
1	S/I	–10	–6	–4	10.4	7.3	5.0
	R/L	3	3	0
	A/P	0	3	–3
2	S/I	0	0	0	0.0	3.2	3.2
	R/L	0	3	–3
	A/P	0	1	–1
3	S/I	0	0	0	8.1	6.7	4.5
	R/L	4	6	–2
	A/P	7	3	4
4	S/I	0	0	0	10.4	7.0	4.2
	R/L	3	0	3
	A/P	–10	–7	–3
5	S/I	0	0	0	2.8	4.5	2.0
	R/L	2	2	0
	A/P	–2	–4	2
6	S/I	0	0	0	2.8	4.1	2.2
	R/L	2	1	1
	A/P	2	4	–2
7	S/I	0	2	–2	3.6	4.1	2.4
	R/L	2	3	–1
	A/P	3	2	1
8	S/I	10	6	4	11.4	8.4	10.8
	R/L	2	3	–1
	A/P	–5	5	–10
9	S/I	–5	0	–5	7.7	2.0	6.6
	R/L	–5	–2	–3
	A/P	−3	0	−3
10	S/I	0	5	–5	2.0	6.2	5.8
	R/L	0	–3	3
	A/P	2	2	0
				avg	5.9	5.3	4.7
						sd	2.7

The shift vector **b** depends on the random day‐to‐day shifts of the bony landmarks with respect to the skin marks. If the skin marks are “optimally” placed, the daily shift vector **b** will be in all directions and its magnitude frequently small. If there is a systematic error, e.g., because the patient was tense during the initial CT scan, **b** will be relatively large and in the same general direction every day. The same is true for the shift vector **s**. The prostate shifts in relation to the bony landmarks from day to day, and depending on where it was “caught” on the initial scan, the shift vectors will be either random and often short, or relatively large and in the same general direction.

**Figure 4:**
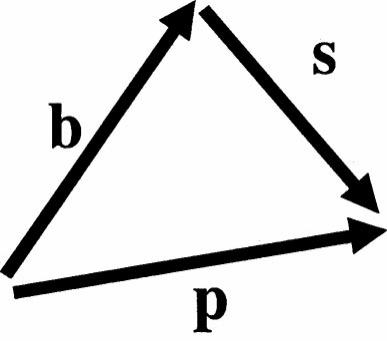
Relative magnitudes and directions of typical vectors **p**, **b**, and **s**.

With ten patients we had 10×3(orthogonal directions)=30 sets of shift coordinates. We found the Primatom CT shifts $p_{i}$ significantly correlated with the bony landmark shifts $b_{i}$, with a correlation coefficient of 0.71 and p<0.001 with Fisher Z test. This demonstrated that, for individual patient, the shifts based on bony anatomy were often in the general direction as the true shifts indicated by Primatom CT, meaning the angle between the two shifts is less than 90°. However, the shortfalls of bony structure, $s_{i}$, had also a high correlation coefficient of 0.65 and p<0.001 with $p_{i}$. That meant adjustments with bony landmarks frequently did not go far enough and substantial positional differences existed between treatments guided by Primatom CT versus bony images. The average radial distances of the shifts **p**, **b**, and **s**, were 5.9, 5.3, and 4.7 mm, respectively (Fig. 4). The magnitudes of **p** and **b** were in line with other studies.^3^ The fact that **s**, prostate organ motion relative to the bones, had an average length of 4.7 mm (with standard deviation of 2.7 mm) showed that bony localization was far from perfect and that FOCAL therapy with treatment CT represented a significant improvement over port filming.

## DISCUSSION

**Figure 5:**
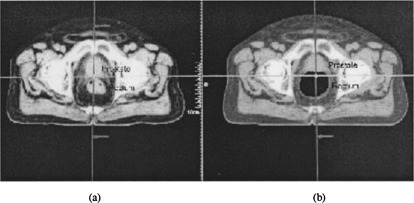
(Color) A prostate patient. (a) was from the planning CT, while (b) was from the treatment CT. The portal images did not indicate any movement, but the CT images showed clearly that the rectal content had changed significantly the posterior border of prostate relative to the isocenter.

CT‐based conformal and intensity modulated radiation therapy represents a big advance on the accuracy of treatment planning. It calls for a corresponding improvement in treatment localization for the patients to benefit fully from this revolution. The prostate organ is known to have substantial motion during radiation treatment.^1^ ^–^ ^3^ Motion uncertainty may be classified as inter‐fractional (between fractions on different days) or intra‐fractional (within the same fraction), systematic (which may be corrected by one single isocenter shift for all fractions) or random (which cannot be corrected by one single isocenter shift), and set‐up uncertainty (motion of bony structure) or organ motion (relative to bony structure). Figure 5 shows an example of prostate motion relative to surrounding pelvic bones. (Figure 5a) was from the planning CT, while Fig. 5(b) was from the treatment CT The simulated DRR port films did not indicate any movement, but the CT images showed clearly that the rectal content had changed significantly the posterior border of prostate relative to the isocenter.

A geometrical miss with portal imaging may result in cold spots in the PTV, i.e., regions with delivered dose less than the prescription. A low dose region increases the risk of local recurrence of tumor. A geometrical miss may also deliver more radiation to the critical organs, with higher complication rate as a consequence. Much effort has been spent on finding a way to better verify the prostate position during treatment. Several methods represent a step forward from portal imaging, and are also valid in studying organ motion. One technique is by implanting radio‐opaque markers inside.^4^ However, this is an invasive procedure, and assumptions have to be made that the marker does not move relative to the prostate organ, and that the prostate does not change shape or size. Another method to visualize the prostate is with an ultrasound probe.^5^ Nevertheless, ultrasound images of prostate are less well defined, may be interpreted differently from CT images, and are not easily applicable for other disease sites.

We are careful in using the term treatment “localization” and not treatment “verification.” Traditionally “verification” means obtaining an image during the whole time of x‐ray exposure of a treatment fraction. CT imaging at the linear accelerator provides a snapshot and hence is not “verification.”

Uncertainty in treatment delivery limits the usefulness of CT planning, and we propose that CT localization is the best solution. The advantages of CT localization are (1) it is noninvasive; (2) image of the entire prostate organ is obtained in one scan; (3) it avoids difference in interpretation since planning and treatment localization are both with CT; (4) the technique is applicable for many disease sites. Improved localization allows for tighter planning target volume margin around gross tumor, and should lead to reduce toxicity to critical organ. In the case of prostate, we decrease the posterior PTV margin around the CTV to 5 mm, which should reduce rectal complication. Even with CT scanning, one does not capture all the motion, notably the random intra‐fractional motion. This is particularly true for organs affected by breathing motion, and may require extra kinds of immobilization, such as an active breathing control device.^10^

Our prostate locations were mapped on the BEV printout manually. This prolonged the time needed in treatment, and each FOCAL treatment fraction took an average of 30 min. This compared with 20 min for a treatment with port film localization. This makes the time delay between localization imaging and treatment delivery longer for CT than for portal imaging. Therefore, we recommend CT over portal imaging only when the inter‐fractional organ motion is larger than the intra‐fractional, as is the case for prostate.^1^ ^–^ ^4^ Our manual registration method contributed to the prolonged time, and automatic image registration software should be able to speed up the process.

## CONCLUSION

Treatment localization based on portal images and bony structure usually shows shifts in the general directions of the real shifts. However, our study illustrates that organ motion relative to surrounding bone is significant, and shows the superiority of FOCAL treatment guided by Primatom CT versus portal image localization.
